# Goats in the city: prevalence of *Giardia duodenalis* and *Cryptosporidium* spp. in extensively reared goats in northern India

**DOI:** 10.1186/s13028-017-0354-4

**Published:** 2017-12-22

**Authors:** Kjersti Selstad Utaaker, Nina Myhr, Rajinder Singh Bajwa, Himanshu Joshi, Anil Kumar, Lucy J. Robertson

**Affiliations:** 1 Parasitology Laboratory, Department for Food Safety and Infection Biology, Faculty of Veterinary Medicine, Norwegian University of Life Sciences, Adamstuen Campus, PO Box 8146, Dep. 0033 Oslo, Norway; 2 Veterinary Hospital for Large Animals, Sector 38, Chandigarh, 160036 India; 3 Department of Medical Parasitology, Postgraduate Institute of Medical Education and Research, Chandigarh, 16002 India

**Keywords:** Backyard livestock, *Cryptosporidium* spp., Low-income countries, *Giardia duodenalis*, Goat, One health, Zoonosis

## Abstract

**Background:**

Various characteristics of goats mean they are highly suitable livestock for backyard rearing by people with limited resources. They are a popular livestock choice in India, where they are often kept to supplement an already scarce income. In these settings, hygiene and sanitation standards tend to be low, and weakens the interface between humans and animals, thus reducing the barrier between them and thereby increasing the likelihood that zoonotic and anthroponotic infections will occur.

**Results:**

This study reports an investigation of the occurrence of *Cryptosporidium* spp. and *Giardia duodenalis* in goats being reared in different settings in urban and peri-urban areas in northern India, and addressed the zoonotic potential of these important protozoan parasites shed from goats living close to humans. The overall prevalence of *G. duodenalis* was 33.8 and 0.5% for *Cryptosporidium* spp.; the relatively low prevalence of cryptosporidiosis may reflect that most samples were derived from adult animals. The prevalence of *G. duodenalis* excretion was found to be similar to that reported in other studies. However, although other studies have reported a predominance of non-zoonotic Assemblage E in goats, in this study potentially zoonotic Assemblages predominated [Assemblage A (36%) and Assemblage B (32%)].

**Conclusions:**

The results of this study indicate that in this area where goats and humans are living in close proximity, there may be sharing of intestinal parasites, which can be detrimental for both host species.

**Electronic supplementary material:**

The online version of this article (10.1186/s13028-017-0354-4) contains supplementary material, which is available to authorized users.

## Background

The potential for transmission of zoonotic agents between humans and animals is amplified when they live in close proximity and the hygienic setting is poor. Backyard livestock are often relied upon to provide extra income or food. Goats are highly suitable for backyard rearing for people with limited resources, as their grazing preferences enable them to feed on plants that other domestic animals refuse, their small size require less space than larger animals, and they are cheaper to buy and maintain [1]. Unlike sheep, goats have a high capacity for adapting to extreme climatic conditions, and are therefore particularly valuable in arid and semi-arid regions. Although sheep are more common than goats on a global scale, in India the goat population is more than double the sheep population, being 154 million goats and 63 million sheep in 2014 [2, 3].

*Cryptosporidium* spp. and *Giardia duodenalis* are among the most common enteric parasites of domestic animals, humans and wildlife [4]. They are two of the most common aetiological agents of paediatric diarrhoea in low-income countries, and are associated with elevated mortality as well as morbidity in this age group [5, 6]. Given the high prevalence of giardiosis and cryptosporidiosis in people living in underdeveloped communities, these diseases were included in the WHO “neglected disease initiative” in 2004 [7].

Studies from India have shown that human giardiosis is prevalent throughout the country, with prevalence rates from northern India ranging from 5.5 to 70%, with highest rates in low socioeconomic groups in Chandigarh [8]. Giardiosis has a significant public health impact and the potential effect of *G. duodenalis* on growth and cognitive functions of children, particularly in low-income countries, where people are exposed to other insults to their health, is particularly important [9]. *G. duodenalis* is also a common infection in animals, and is sometimes associated with disease [10–12]. Some *G. duodenalis* Assemblages are apparently host-specific, while others are less so [13]. In northern India, one survey of human infections reported Assemblage B to be most common, which is usually associated with anthroponotic transmission [14]. Studies on *G. duodenalis* infections in goats are relatively rare, but a review from 2009 suggested a prevalence of around 20%, with most genotyped isolates from goats being genotype E, which is not zoonotic [15].

Cryptosporidiosis can be caused by several species and genotypes of *Cryptosporidium* [16]. In humans, *C. hominis* and *C. parvum* are the aetiological agents responsible for most infections [17]; *C. hominis* largely infects humans whereas zoonotic *C. parvum* primarily infects ruminants and humans. *Cryptosporidium* spp. prevalences reported in India range from 3.8% in patients in northern India, with the majority of infections attributed to *C. hominis* [18], to 39.7% in rural populations in southern India [19]. Again, studies on this parasite in goats are not very common, but a review estimated an approximate global prevalence of around 15% [15].

However, few studies have investigated the prevalence of these infections in locations where the potential for transmission between goats and their owners is greatest, and where they are most likely to exert the greatest impact on each other [15, 20].

The aim of this study was to determine the prevalence and zoonotic potential of *Cryptosporidium* spp. and *G. duodenalis* in small-scale goat farms and backyard livestock goats in urban and peri-urban areas in northern India.

## Methods

### Sampling

In February 2016, a total of 207 faecal samples from 207 individual goats held in 16 separate goat holdings in Chandigarh, Punjab, and Haryana were collected. The samples were collected from both urban “village” areas in Chandigarh, and peri-urban villages in the neighbouring states of Punjab and Haryana. These goats were mainly kept for meat production. The goats were housed in and around human settlements, either in a simple shed adjoining their owners’ home or in a pen actually under the same roof as their owners. The goats were taken out during daytime for grazing in the nearby environment. The hygiene status was fair in most of the pens, and manure was cleaned out at least once a day. The goats were mainly tended by the families, particularly women and children, which owned them.

Singha Devi, Jayenti and Kurali are small peri-urban towns and villages located in the S.A.S Nagar district in the state Punjab, and Saketri is a peri-urban village in the Panchkula district of the Haryana state. The city Chandigarh has a population of about one million, with almost all of its inhabitants living in urban areas. The population density in S.A.S Nagar and Haryana is approximately ten times lower than in Chandigarh [21], and S.A.S Nagar and Panchkula have an approximately ten times higher goat livestock than Chandigarh [22–24].

The city Chandigarh has a population of about one million, with almost all of its inhabitants living in urban areas. Kansal and Maloya are so-called non-sectorial villages associated with the city, but with poorer infrastructure and lower socioeconomic levels compared with the city itself [25]. Sector 38 West is the location of the slum colony Rajiv. Slum settlements have grown in the past decades in Chandigarh, especially in the periphery of the city, where poor families tend to settle due to cheap housing. It was in these areas that goats were kept. The goat population of Chandigarh has been estimated to be 805 [24].

**Fig. 1:**
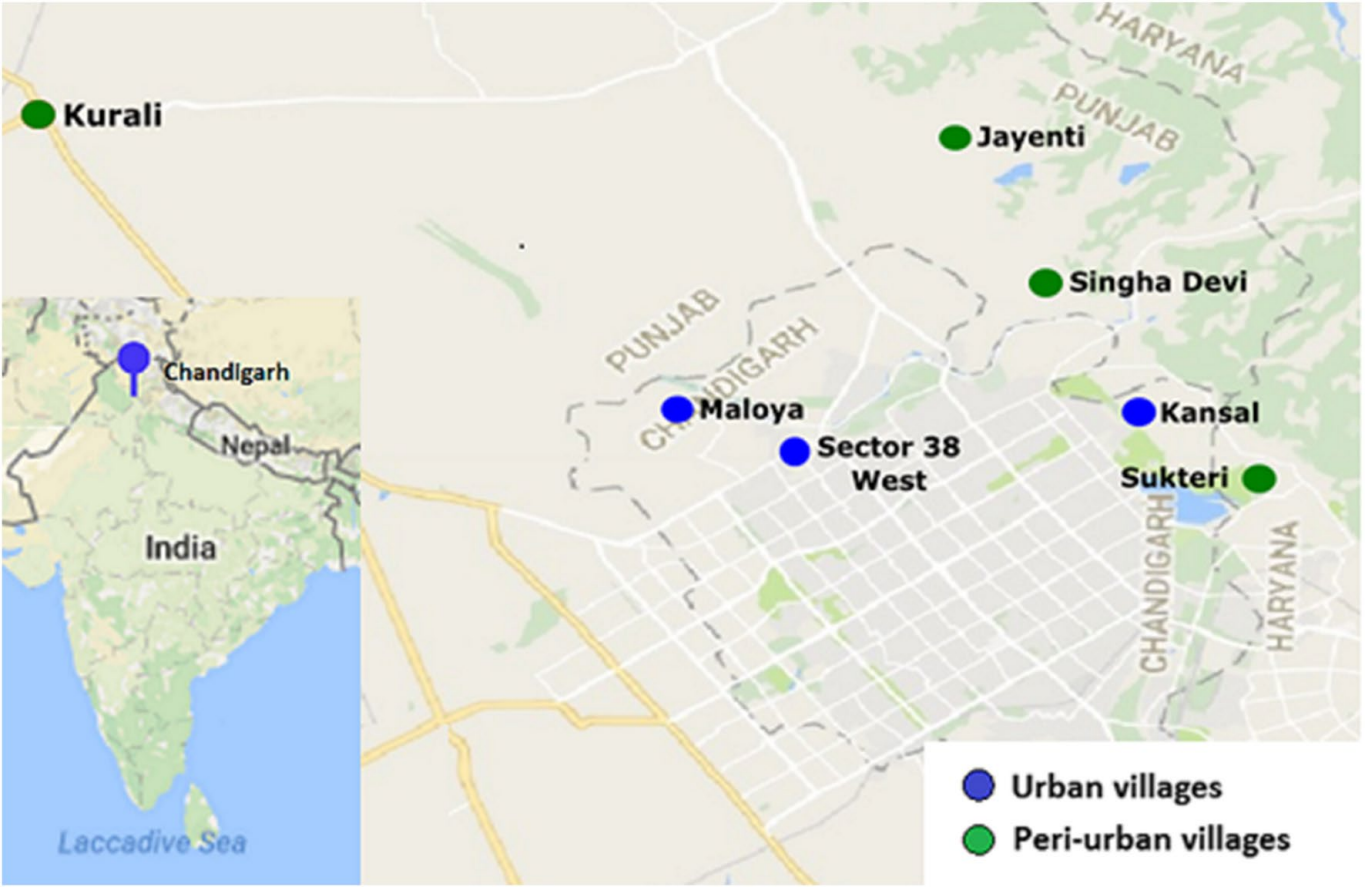
Areas where samples were collected

Goat owners in these areas were contacted by visits and those agreeing to contribute samples were included in the study. The goats kept in this area are mainly of the Beetal breed, and the number of animals ranged from 2 to 29 from the 16 herds included. The samples were obtained from flocks in which the majority of animals were adults, as based on body size and weight estimation of the animals. The faecal samples were firm and pelleted, and there were no signs of diarrhoea. The samples (each approximately 5–10 g) were collected either rectally or non-invasively promptly after defecation, and were immediately mixed with 2.5% potassium dichromate and stored at 4 °C before shipping to the Parasitology Laboratory, Norwegian University of Life Sciences (NMBU) for analysis. The ages of the goats were not recorded, but the majority were adults (Fig. 1).

### Analysis of goat faeces for *Cryptosporidium* spp. oocysts and *G. duodenalis* cysts

The samples were analysed by immunofluorescent antibody (IFAT) staining for the presence of *G. duodenalis* cysts and/or *Cryptosporidium* spp. oocysts, either in direct faecal smears or following immunomagnetic separation (IMS). This method comparison was conducted in order to determine whether one method was more sensitive than the other, and the more sensitive method would be used for the remaining samples.

### Immunomagnetic separation before IFAT analysis

All faecal samples (n = 207) were washed with phosphate buffered saline, and then passed through a faecal parasite concentrator with a pore diameter 425 μm (Midi Parasep, Apacor, Berkshire, UK) and centrifuged to create a pellet. *G. duodenalis* cysts and *Cryptosporidium* spp. oocysts were isolated from 200 µL of concentrated faeces using an in-house immunomagnetic separation method (IMS) using Dynabeads^®^(GC-Combo, Life Technologies, Carlsbad, CA) [26, 27]; 10 μL anti-*G. duodenalis* beads, 10 μL anti-*Cryptosporidium* spp. beads, 80 μL Sur-Modics StabilZyme^®^, 20 μL SL Buffer B and 100 µL Buffer Q4 were used to generate 55 μL of purified sample from approximately 200 mg of the faecal pellet. 5 μL of the resulting purified sample was dried and fixed with methanol to multispot welled slides for detection of *G. duodenalis* cysts and *Cryptosporidium* spp. oocysts using a *Cryptosporidium/Giardia* direct IFAT; Aqua-Glo, Waterborne Inc., New Orleans), in accordance with manufacturer’s instructions. Prior to being screened, samples were also stained with 4′6 diamidino-2-phenylindole (DAPI), a non-specific fluorescent stain that binds to double-stranded DNA.

### Faecal smear preparation before IFAT analysis

Homogenized and sieved faecal material (5–20 µL) from 40 randomly selected samples were placed on a microscope slide using plastic bacteriological loops that take approx. 10 µL amount of sample. The samples were left to dry and then fixed with methanol before staining with 15 µL of monoclonal antibody and incubation as described for IMS. DAPI staining was not used in this preparation due to the amount of other DNA from other debris in faecal smears.

After fixing and staining, preparations from both faecal smears and IMS were screened under a fluorescent microscope with the following filter settings: FITC: emission-490 nm, excitation-525 nm and DAPI: emission-350 nm, excitation-470 nm.

The number of cysts/oocysts per field of view at objective × 20 were enumerated and samples were graded according to Table 1. For samples in which IMS was used prior to IFAT, the number of cysts/oocysts in the final concentrate were enumerated, and the data used to estimate the number of cysts/oocysts per gram faeces.

**Table 1:** Grading of *Giardi*a *duodenalis* cyst and *Cryptosporidium* spp. oocyst counts visualized using immunofluorescent microscopy

Oocyst/cyst count	Grading
1–9	+
10–50	++
51–100	+++
> 100	++++

## Molecular methods

### DNA extraction

The contents of each microcentrifuge tube containing *Cryptosporidium* spp. oocysts and *G. duodenalis* cysts were re-suspended in Tris–EDTA buffer and held at 100 °C for *Cryptosporidium* spp. oocysts and 90 °C for *G. duodenalis* cysts for 1 h, before the DNA was isolated using QIamp DNA mini kit (Qiagen GmbH), using an overnight step at 56 °C.

### PCR, electrophoresis, purification of PCR product, and sequencing

Samples that were DAPI–positive were selected for genotyping and polymerase chain reaction (PCR) analysis, regardless of number of (oo)cysts.

Four genes were used for genotyping investigations of the *G. duodenalis* DAPI-positive samples by conventional PCR; the β-giardin gene, the glutamate dehydrogenase (*gdh*) gene, the triosephosphate isomerase (*tpi*) gene, and the small subunit ribosomal RNA (SSU RNA) gene. For the sample with *Cryptosporidium* spp. oocysts, primers targeting sections of the genes SSU rRNA, COWP, and Actin were used, also by conventional PCR. The primers and reaction cycles are further described in Additional file 1.

For all genes, the following PCR mixture was used: 10 pmol of each primer (1 µL), 0.4 μL of bovine serum albumin (20 mg/mL), 5.8 μL of water, 25 μL of HotStartTaqmaster (QIAGEN^®^ GmbH, Germany), and 2 μL of template. For each set of reactions, a negative control (2 µL water) and a positive control (2 µL DNA from *G.* *duodenalis* H3 isolate belonging to assemblage B, Waterborne Inc., New Orleans, USA, or *C. parvum* oocysts, with species identification by Hønsvall and Robertson [28]) were included, and the total volume of each reaction was 25 µL.

PCR products were electrophoresed on 1% agarose gels and stained with SYBRsafe^®^ DNA gel stain under UV radiation. Positive samples were purified using ROCHE^®^ high pure PCR product purification kit, and purified products were sent along with appropriate primers for sequencing on both strands at GATC Biotech, Germany. Sequences were examined using Geneious 10.1.2 software and sequence comparisons conducted using NCBI BLAST.

Sequences were submitted to GenBank and the Accession numbers are provided in the results.

### Statistics

The two preparation methods, IMS and smear before IFAT, were analysed using 40 randomly selected samples and compared using Fisher’s exact test (GraphPad Software, Inc.), based on categorical data in a two-by-two contingency table.

For comparison of *G. duodenalis* prevalence according to location (urban/peri-urban), Chi square test was used (MediCalc Software bvba). Similar comparisons for *Cryptosporidium* spp. were not conducted, due to low prevalence.

## Results

### Comparison of faecal smears and IMS for detection of cysts

A comparison of the two faecal examination methods is shown in Table 2. IMS prior to IFAT staining detected significantly more positive samples than preparing a faecal smear before staining (P < 0.001; Table 2).

**Table 2:** Contingency table, results of Fisher’s exact test

	Smear		Total
	Positive	Negative	
IMS			
Positive	12	13	25
Negative	0	15	15
Total	12	28	n = 40

### Prevalence of *Cryptosporidium* spp. and *Giardia duodenalis*

Examination of the faecal samples using the IMS and IFAT protocol revealed the presence of *G. duodenalis* cysts in 33.8% (70/207) of samples and *Cryptosporidium* spp. in 0.5% of samples (Table 3). All samples that were smear-positive were also positive when IMS was used prior to staining.

**Table 3:** Overall prevalence of *Giardia duodenalis* and *Cryptosporidium* spp. in goats according to area of sampling

Place	No of goats sampled	*Giardia* positive	*Cryptosporidium* positive
Chandigarh (urban)			
Kansal (3 herds)	71	13	–
Sector 38 west (5 herds)	30	18	–
Maloya (1 herd)	4	1	–
Summary urban samples	105	32	
Punjab (peri-urban)			
Jayenti (3 herds)	29	9	–
Singha Devi (1 herd)	10	1	1
Kurali (1 herd)	20	10	–
Haryana (peri-urban)			
Saketri (2 herds)	43	18	–
Summary peri-urban samples	102	38	
Total	207	70	1

– not detected

The prevalence in the urban and peri-urban areas was 30.5% (32/105) and 37.3% (38/102), respectively. These proportions were not statistically different (P > 0.05).

### Intensity of shedding of *Cryptosporidium* spp. and *Giardia duodenalis*

Of the *G. duodenalis*-positive samples, most (75%) had a low to moderate (+ and ++) number of cysts, and 25% had a high number of cysts (+++ and ++++) (Table 4). From 55 to over 55,000 cysts per gram faeces (Mean: 8671, Median: 275) were found.

**Table 4:** Intensity of infection from positive samples and sampling area according to immunomagnetic separation results

Intensity of infection	G+	G++	G+++	G++++	C++
Chandigarh					
Kansal	10	2	–	–	–
Sector 38 west	8	2	1	8	–
Maloya	1	–	–	–	–
Punjab					
Jayenti	6	3	–	–	–
Singha Devi	1	–	–	–	1
Kurali	5	–	–	5	–
Haryana					
Saketri	9	6	–	4	–
Total	40	13	1	17	1

– not detected

The *Cryptosporidium* spp.-positive samples had moderate (++) oocyst excretion (Table 4).

### Molecular analyses

The PCR at different genetic loci had the following sensitivities: SSU 50% (26/52), Beta-giardin 1.9% (1/52), TPI 5.7% (3/52), and GDH 9.6% (5/52).

PCR and sequencing on the single *Cryptosporidium* spp.-positive sample revealed *C. ubiquitum* (GenBank Accession number: MF124820).

An overview of the *G. duodenalis* genotyping results is provided in Table 5. Based on all the results combined from the different PCR, the majority (68%) of *G. duodenalis* Assemblages identified were potentially zoonotic (A or B), with 10 out of 28 (36%) genotyped samples Assemblage A, 9 (32%) Assemblage B, 8 (29%) Assemblage E, 1 and one (4%) Assemblage D. One of the samples was sequenced to be Assemblage E at the GDH gene, and Assemblage C at the SSU gene.

**Table 5:** Results from sequencing and analysis of positive polymerase chain reaction products. *Giardia* assemblages are noted with capital letter before semicolon preceding GenBank Accession number

Area	Sample no	No. of cysts isolated by IMS^a^	DAPI (%)^b^	GDH^c^	TPI^d^	Bg^e^	SSU^f^
Chandigarh							
Kansal	1	10	100	–	–	–	B; MF069062
	2	220	30	–	–	–	E; MF069058
	3	10	100	–	–	–	A; MF069057
	4	20	100	–	–	–	B; MF069047
	5	100	40	–	–	–	A; MF069052
	6	10	100	–	–	–	A; MF069051
Sector 38 west							
	7	1000	70	E; MF084938	–	–	C; MF069071
	8	10,000	5	E; MF084935	–	–	E; MF069070
	9	10	100	–	–	–	A; MF069056
	10	10,000	90	–	–	–	E; MF069059
	11	100	60	–	–	–	D; MF069055
Punjab							
Singha Devi							
	12	30	67	–	–	–	A; MF069054
Kurali							
	13	10,000	90	E; MF084936	E; MF095054	E; MF106203	E; MF069072
	14	10,000	70	E; MF084934	E; MF095052	–	–
	15	10,000	80	–	B; MF095053	–	B, MF069053
	16	20	100	–	–	–	B; MF069066
	17	20	50	–	–	–	B; MF069064
Jayenti							
	18	10	100	–	–	–	B; MF069060
Haryana							
Saketri							
	19	100	10	E; MF084937	–	–	–
	20	50	60	–	–	–	B; MF069050
	21	30	30	–	–	–	A; MF069068
	22	100	40	–	–	–	A; MF069067
	23	1000	90	–	–	–	E; MF069065
	24	40	25	–	–	–	B; MF069063
	25	20	50	–	–	–	B; MF069061
	26	120	33	–	–	–	A; MF069069
	27	500	44	–	–	–	A; MF069049
	28	160	87	–	–	–	A; MF069048

PCR conditions and reaction times can be found in Additional file 1

– no amplification

*TPI* triosephosphate isomerase; *GDH* glutamate dehydrogenase; *BG* beta giardin; *SSU* small subunit; rRNA; − PCR Negative; Assemblage (GenBank Accession number) where sequence of PCR products was obtained

^a^Number of *Giardia* cysts used for DNA isolation

^b^proportion of DAPI positive *Giardia* cysts used for DNA isolation

^c^Additional file 1; Ref. [4]

^d^Additional file 1; Ref. [3]

^e^Additional file 1; Ref. [7]

^f^Additional file 1; Ref. [1, 2]

## Discussion

The main finding of this cross-sectional survey was that although the prevalence of *Cryptosporidium* spp. infection was low in the samples analysed, the prevalence of infection *with G. duodenalis* was relatively high, and with a preponderance of potentially zoonotic Assemblages. This indicates that goats may be both a reservoir of *G. duodenalis* for human infection, and also may themselves be infected by this parasite excreted from humans.

Reported prevalences for both *G. duodenalis* and *Cryptosporidium* spp. Infection in goats around the globe tend to vary considerably, from 12.3 to 42.2% for *G. duodenalis* infection and from 4.8 to 33.6% for infection with *Cryptosporidium* spp. [29–37]. This might not only reflect the prevalence of infection, but could also be due to variation in the sensitivities of the diagnostic tests used, the age of the goat, and whether only a single or consecutive sample(s) was taken, given the intermittent shedding of *G. duodenalis* cysts, and the acute nature of cryptosporidiosis. The low prevalence of infection with *Cryptosporidium* spp. in our study probably reflects that most samples were derived from adult animals; although some species of *Cryptosporidium* (*C. xiaoi* and *C. ubiquitum*) tend to be associated with slightly older age groups of goats, in general *Cryptosporidium* spp. are recognized to primarily infect goat kids, due to the development of immunity [38].

When determining whether *G. duodenalis* and *Cryptosporidium* spp. infections in animals may be of relevance in a public health context, identifying the species and genotypes involved is imperative.

The SSU rRNA marker, which had the highest sensitivity in this study, is commonly used for assemblage differentiation of *G. duodenalis* assemblages, but might be insufficient for confident identification of the assemblage due to low levels of phylogenetic resolution, perhaps related to its multi-copy nature [39].

Finding *G. duodenalis* from Assemblage B in goats is rather unusual compared with other studies; a review from 2009 reports that Assemblage E is most frequently reported—with potentially zoonotic infection, particularly with Assemblage B, occurring relatively rarely [15]. The difference between our study and most other studies is the close contact between the goats being sampled and the human–environment in our study. The proximity of humans and goats in our study area, along with the supporting evidence for molecular results, might suggest zoonotic/anthropozoonotic spread of the parasite in such situations.

One sample was sequenced as assemblage D, which is a canid-specific genotype. As this sample had low numbers of cysts it seems likely that this represents carriage from the goat ingesting cysts from dog faeces and then excreting them, rather than infection. This may also be the case where Assemblage E was indicated from PCR at one gene, and Assemblage C at another. Whether this may apply to other samples cannot be determined.

The grazing habits of goats, generally browsing on woody shrubs and weeds rather than grazing grass may indicate that they are less likely to ingest parasites [15]. However, in urban or peri-urban settings where shrubs are scant, they will be forced to search for nutrients closer to the ground, thus being more likely to ingest *G. duodenalis* cyst or *Cryptosporidium* spp. oocysts contaminating the environment.

One sample contained *Cryptosporidium ubiquitum*; this species has been found in a wide range of animals as well as humans [40], and thus represents zoonotic and anthroponotic potential, especially in the setting of a shared household between goats and humans in lower socioeconomic areas. A vast amount of epidemiological data demonstrates strong links between contact with infected livestock and human infections [4]; as most of the goats in our study were living close to humans, often sharing the same household, sharing of intestinal parasites would not be very surprising.

There was no significant difference between the samples collected in urban and peri-urban areas, and the genotyping results showed an even spread of the *G*. *duodenalis* genotypes in the given areas. This was unexpected, as we had hypothesised that goats in urban areas might be more exposed to human genotypes than the peri-urban flocks.

However, contamination of the environment by human faeces is common everywhere, and in the urban setting people may be more likely to use a latrine for defecation than in peri-urban areas, where open defecation is known to be common.

Although using IMS for analysis of faecal samples is more time-consuming and expensive our results indicate that it was more sensitive; this is presumably due to the larger quantity of faecal matter than can be analysed and also, perhaps, due to less debris in the sample. In cleaner samples, it is also easier to determine the suitability of the sample for molecular analyses based on DAPI-staining due to there being lower background fluorescence. This method could be a useful tool for other field studies where it is only possible to obtain a single sample per animal, not the three consecutive ones which are recommended due to the intermittent shedding of cysts to obtain a more certain answer of the true prevalence.

Infected livestock have long been suggested as sources for contaminating food and water in outbreaks, but molecular analyses has often incriminated human effluent as the source [4]. Nonetheless, as an adult goat produces between 1 and 3 kg of faeces on a daily basis, it is clear that the potential for environmental contamination is considerable [15], especially when the animals are kept on a free-range basis in a community where the overall density is high. The common characteristics of *G. duodenalis* and *Cryptosporidium* spp., having a low infectious dose, (oo)cysts being infective immediately after excretion, and their robustness enabling them to survive for months in the environment [40], are epidemiological traits well-suited for causing infectious foci in places with high population densities and extensive animal husbandry.

In addition, a serious constraint to economical and intensive goat production is the mortality of kids as a result of diarrhoea up to the age of 3 months [3], and among the pathogens causing the diarrhoea, *Cryptosporidium* spp. is principally involved [36, 41]. *G. duodenalis* infection in ruminants is, on the other hand, often asymptomatic, but may also be associated with the occurrence of diarrhoea and ill-thrift [42], which may lead to economic losses as well as reduced welfare of the flock.

## Conclusions

Goats were frequently found to harbour zoonotic genotypes of *G. duodenalis*. Previous studies have found *G. duodenalis* infections in small ruminants to belong to non-zoonotic assemblages, and thus goats have not previously been considered to be a reservoir of infection for *G. duodenalis* in humans. Our results may reflect that in this situation goats live in closer contact with their owners than in the majority of other published studies. As keeping goats in low-income countries is often a trade for the poorest in society, the awareness of One Health for one household through proper hygienic routines and animal management could be of benefit for both human and animal health, as well as improving both the economy and husbandry of the goat keepers and their herds.

## Additional file

**Additional file 1: Table S1.** Polymerase chain reaction conditions for detection of *Giardia* and *Cryptosporidium.*

## References

[1] Pollott G, Wilson RT. Sheep and goats for diverse products and profits. FAO diversification booklet. 2009;9:42. http://www.fao.org/3/a-i0524e.pdf Accessed 4 Dec 2017.

[2] Food and Agriculture Organization of the United Nations. Faostat. http://www.fao.org/faostat/en/#data/QA Accessed 29 July 2017.

[3] Paul S, Sharma DK, Boral R, Mishra AK, Nayakwadi S, Banerjee PS, Pawaiya RS (2014). Cryptosporidiosis in goats: a review. Adv Anim Vet Sci.

[4] Thompson RCA, Palmer CS, O’Handley R (2008). The public health and clinical significance of *Giardia* and *Cryptosporidium* in domestic animals. Vet J.

[5] Kotloff KL, Nataro JP, Blackwelder WC, Nasrin D, Farag TH, Panchalingam S (2013). Burden and aetiology of diarrhoeal disease in infants and young children in developing countries (the Global Enteric Multicenter Study, GEMS): a prospective, case-control study. Lancet.

[6] Platts-Mills JA, Babji S, Bodhidatta L, Gratz J, Haque R, Havt A (2015). Pathogen-specific burdens of community diarrhoea in developing countries: a multisite birth cohort study (MAL-ED). Lancet Glob Health.

[7] Savioli L, Smith H, Thompson A (2006). *Giardia* and *Cryptosporidium* join the ‘neglected diseases initiative’. Trends Parasitol.

[8] Laishram S, Kang G, Ajjampur SSR (2012). Giardiasis: a review on assemblage distribution and epidemiology in India. Indian J Gastroenterol.

[9] Berkman DS, Lescano AG, Gilman RH, Lopez SL, Black MM (2002). Effects of stunting, diarrhoeal disease, and parasitic infection during infancy on cognition in late childhood: a follow-up study. Lancet.

[10] Farthing MJG (1997). The molecular pathogenesis of giardiasis. J Pediatr Gastroenterol Nutr.

[11] Buret AG (2007). Mechanisms of epithelial dysfunction in giardiasis. Gut.

[12] Geurden T, Claerebout E, Dursin L, Deflandre A, Bernay F, Kaltsatos V, Vercruysse J (2006). The efficacy of an oral treatment with paromomycin against an experimental infection with *Giardia* in calves. Vet Parasitol.

[13] Ryan U, Cacciò SM (2013). Zoonotic potential of *Giardia*. Int J Parasitol.

[14] Ghoshal U, Shukla R, Pant P, Ghoshal UC (2016). Frequency, diagnostic performance of coproantigen detection and genotyping of the *Giardia* among patients referred to a multi-level teaching hospital in northern India. Pathog Glob Health..

[15] Robertson LJ (2009). *Giardia* and *Cryptosporidium* infections in sheep and goats: a review of the potential for transmission to humans via environmental contamination. Epidemiol Infect.

[16] Xiao L, Feng Y (2008). Zoonotic cryptosporidiosis. FEMS Immunol Med Microbiol.

[17] Xiao L, Fayer R, Ryan U, Upton SJ (2004). *Cryptosporidium* taxonomy: recent advances and implications for public health. Clin Microbiol Rev.

[18] Sharma P, Sharma A, Sehgal R, Malla N, Khurana S (2013). Genetic diversity of *Cryptosporidium* isolates from patients in North India. Int J Infect Dis..

[19] Kang G, Mathew MS, Rajan DP, Daniel JD, Mathan MM, Mathan VI, Muliyil JP (1998). Prevalence of intestinal parasites in rural Southern Indians. Trop Med Int Health..

[20] Daniels ME, Shrivastava A, Smith WA, Sahu P, Odagiri M, Misra PR (2015). *Cryptosporidium* and *Giardia* in humans, domestic animals, and village water sources in rural India. Am Trop Med Hyg..

[21] Population census of India, 2011. Government of India. http://www.census2011.co.in/census/state/chandigarh.html. Accessed 30 July 2017.

[22] Brief industrial profile of district S.A.S NAGAR, micro, small and medium enterprises development institute, Govt of India, Ministry of MSME, www.msmedildh.gov.in, http://dcmsme.gov.in/dips/SAS%20Nagar.pdf. Accessed 30 July 2017.

[23] Department of Animal Husbandry & Dairying, State Government of Haryana, India District wise livestock census 2012; Chapter 14, Haryana, Livestock Population. http://pashudhanharyana.gov.in/html/livestockcensus.html. Accessed 30 July 2017.

[24] Livestock population. Department of Animal Husbandry and Fisheries, Chandigarh Administration. http://chdanimalhusbandry.gov.in/livestock.aspx. Accessed 30 July 2017.

[25] Chandigarh master plan 2031, Demography. http://chandigarh.gov.in/cmp_2031.htm. Accessed 25 Aug 2017.

[26] Utaaker KS, Huang Q, Robertson LJ (2015). A reduced-cost approach for analyzing fresh produce for contamination with *Cryptosporidium* oocysts and/or *Giardia* cysts. Food Res Int.

[27] Robertson L, Hermansen L, Gjerde B, Strand E, Alvsvåg J, Langeland N (2006). Application of genotyping during an extensive outbreak of waterborne giardiasis in Bergen, Norway, during autumn and winter 2004. Appl Environ Microbiol.

[28] Hønsvall BK, Robertson LJ (2017). Real-time nucleic acid sequence-based amplification (NASBA) assay targeting MIC1 for detection of *Cryptosporidium parvum* and *Cryptosporidium hominis* oocysts. Exp Parasitol.

[29] Geurden T, Thomas P, Casaert S, Vercruysse J, Claerebout E (2008). Prevalence and molecular characterisation of *Cryptosporidium* and *Giardia* in lambs and goat kids in Belgium. Vet Parasitol.

[30] Van der Giessen JWB, De Vries A, Roos M, Wielinga P, Kortbeek LM, Mank TG (2006). Genotyping of *Giardia* in Dutch patients and animals: a phylogenetic analysis of human and animal isolates. Int J Parasitol.

[31] Ruiz A, Foronda P, González JF, Guedes A, Abreu-Acosta N, Molina JM, Valladares B (2008). Occurrence and genotype characterization of *Giardia duodenalis* in goat kids from the Canary Islands, Spain. Vet Parasitol..

[32] Castro-Hermida JA, Almeida A, González-Warleta M, da Costa JMC, Rumbo-Lorenzo C, Mezo M (2007). Occurrence of *Cryptosporidium parvum* and *Giardia duodenalis* in healthy adult domestic ruminants. Parasitol Res.

[33] Jafari H, Jalali MHR, Shapouri MSA, Hajikolaii MRH (2014). Determination of *Giardia duodenalis* genotypes in sheep and goat from Iran. J Parasit Dis..

[34] Johnston AR, Gillespie TR, Rwego IB, McLachlan TLT, Kent AD, Goldberg TL (2010). Molecular epidemiology of cross-species *Giardia duodenalis* transmission in western Uganda. PLoS Negl Trop Dis..

[35] Bomfim TCB, Huber F, Gomes RS, Alves LL (2005). Natural infection by *Giardia* sp. and *Cryptosporidium* sp. in dairy goats, associated with possible risk factors of the studied properties. Vet Parasitol.

[36] Noordeen F, Rajapakse R, Faizal ACM, Horadagoda NU, Arulkanthan A (2000). Prevalence of *Cryptosporidium* infection in goats in selected locations in three agroclimatic zones of Sri Lanka. Vet Parasitol.

[37] Delafosse A, Castro-Hermida JA, Baudry C, Ares-Mazás E, Chartier C (2006). Herd-level risk factors for *Cryptosporidium* infection in dairy-goat kids in western France. Prev Vet Med..

[38] Robertson LJ, Björkman C, Axén C, Fayer R. Cryptosporidiosis in farmed animals. In: *Cryptosporidium*: parasite and disease. Berlin: Springer; 2014. p. 149–235.

[39] Feng Y, Xiao L (2011). Zoonotic potential and molecular epidemiology of *Giardia* species and giardiasis. Clin Microbiol Rev.

[40] Fayer R, Santín M, Macarisin D (2010). *Cryptosporidium ubiquitum* n. sp. in animals and humans. Vet Parasitol.

[41] Ershaduzzaman M, Rahman MM, Roy BK, Chowdhury SA (2007). Studies on the diseases and mortality pattern of goats under farm conditions and some factors affecting mortality and survival rates in black Bengal kids. Bangladesh J Vet Med..

[42] O’Handley RM, Olson ME (2006). Giardiasis and cryptosporidiosis in ruminants. Vet Clin N Am Food Anim Pract..

