# Correction to: Goats in the city: prevalence of *Giardia duodenalis* and *Cryptosporidium* spp. in extensively reared goats in northern India

**DOI:** 10.1186/s13028-018-0402-8

**Published:** 2018-09-10

**Authors:** Kjersti Selstad Utaaker, Nina Myhr, Rajinder Singh Bajwa, Himanshu Joshi, Anil Kumar, Lucy J. Robertson

**Affiliations:** 1 Parasitology Laboratory, Department for Food Safety and Infection Biology, Faculty of Veterinary Medicine, Norwegian University of Life Sciences, Adamstuen Campus, PO Box 8146, Dep. 0033 Oslo, Norway; 2 Veterinary Hospital for Large Animals, Sector 38, Chandigarh, 160036 India; 3 Department of Medical Parasitology, Postgraduate Institute of Medical Education and Research, Chandigarh, 16002 India

## Correction to: Acta Vet Scand (2017) 59:86 10.1186/s13028-017-0354-4

In the original publication of this article [1] the supplementary file was missing two primers for the PCR reaction and the PCR conditions of *Giardia* and *Cryptosporidium*. In this correction article the updated additional file (Additional file 1) is available, in which the two primers are included.

## Additional file

**Additional file 1: Table S1.** Updated polymerase chain reaction conditions for detection of *Giardia* and *Cryptosporidium.*
