# Supplementary material for: Correction to: Goats in the city: prevalence of Giardia duodenalis and Cryptosporidium spp. in extensively reared goats in northern India
Source: Acta Vet Scand. 2018 Sep 10;60:52. doi: 10.1186/s13028-018-0402-8 (PMC6130062; doi:10.1186/s13028-018-0402-8)
Supplement: Supplementary file 1 — Additional file 1: Table S1. Updated polymerase chain reaction conditions for detection of Giardia and Cryptosporidium. [file 13028_2018_402_MOESM1_ESM.docx]

**Additional file 1.** Polymerase chain reaction conditions for detection of *Giardia* and *Cryptosporidium.*

| Locus | Amplicon length | Primer | Cycle conditions | | References |
| --- | --- | --- | --- | --- | --- |
| ***Giardia*** |  |  |  |  |  |
| Small Subunit (SSU) rRNA | 292 | 1st amplification | 96 °C, 5 min |  | [1] |
|  |  | F: 5′-CATCCGGTCGATCCTGC-3′ | 96 °C, 30 sec |  | [2] |
|  |  | R: 5′-AGTCGAACCCTGATTCTCCGCCAGG-3′ | 59 °C, 40 sec | 40 x |  |
|  |  |  | 72 °C, 40 sec |  |  |
|  |  |  | 72 °C, 7 min |  |  |
|  |  |  |  |  |  |
|  | 175 | 2^nd^ amplification | 96 °C, 5 min |  |  |
|  |  | F: 5′-GACGCTCTCCCCAAGGAC-3′ | 96 °C, 30 sec |  |  |
|  |  | R: 5′-CTGCGTCACGCTGCTCG-3′ | 55 °C, 40 sec | 50 x |  |
|  |  |  | 72 °C, 30 sec |  |  |
|  |  |  | 72 °C, 7 min |  |  |
|  |  |  |  |  |  |
|  |  |  |  |  |  |
| Triosephosphate Isomerase (TPI) | 605 | 1^st^ amplification | 95 °C, 10 min |  | [3] |
|  |  | F: 5′-AAATYATGCCTGCTCGTCG-3′ | 94 °C, 45 sec |  |  |
|  |  | R: 5′-CAAACCTTYTCCGCAAACC-3′ | 50 °C, 45 sec | 45 x |  |
|  |  |  | 72 °C, 60 sec |  |  |
|  |  |  | 72 °C, 10 min |  |  |
|  |  |  |  |  |  |
|  | 563 | 2^nd^ amplification | 95 °C, 10 min |  |  |
|  |  | F: 5′-CCCTTCATCGGNGGTAACTT-3′ | 94 °C, 45 sec |  |  |
|  |  | R: 5′-GTGGCCACCACVCCCGTGCC-3′ | 50 °C, 45 sec | 45 x |  |
|  |  |  | 72 °C, 60 sec |  |  |
|  |  |  | 72 °C, 10 min |  |  |
|  |  |  |  |  |  |
| Glutamate Dehydrogenase (GDH) |  | 1st amplification: | 94 °C, 15 min |  | [4] |
|  |  | F: 5′-TCAACGTYAAYCGYGGYTTCCGT-3′ | 94 °C, 45 sec |  | [5] |
|  |  | R: 5′-GTTRTCCTTGCACATCTCC-3′ | 54 °C, 45 sec | 50 x |  |
|  |  |  | 72 °C, 45 sec |  |  |
|  |  |  | 72 °C, 10 min |  |  |
|  |  |  |  |  |  |
| Beta Giardin (BG) | 753 | 1st amplification: | 95 °C, 15 min |  | [6] |
|  |  | F: 5′-AAGCCCGACGACCTCACCCGCAGTGC-3′ | 94 °C, 30 sec |  | [7] |
|  |  | R: 5′-GAGGCCGCCCTGGATCTTCGAGAC | 60 °C, 30 sec | 35 x |  |
|  |  | GAC-3′ | 72 °C, 60 sec |  |  |
|  |  |  | 72 °C, 10 min |  |  |
|  |  |  |  |  |  |
|  | 511 | 2nd amplification | 95 °C, 15 min |  |  |
|  |  | F: 5′-GAACGAGATCGAGGTCCG-3′ | 95 °C, 30 sec |  |  |
|  |  | R: 5′-CTCGACGAGCTTCGTGTT-3′ | 53 °C, 30 sec | 40 x |  |
|  |  |  | 72 °C, 60 sec |  |  |
|  |  |  | 72 °C, 10 min |  |  |
|  |  |  |  |  |  |
| ***Cryptosporidium*** |  |  |  |  |  |
| SSU rRNA | 860 | amplification | 95 °C, 15 min |  | [8] |
|  |  | F: 5′-GGAAGGGTTGTATTTATTAGATAAAG-3′ | 94 °C, 45 sec |  |  |
|  |  | R: 5′-AAGGAGTAAGGAACAACCTCC A-3′ | 55 °C, 45 sec | 50 x |  |
|  |  |  | 72 °C, 60 sec |  |  |
|  |  |  | 72 °C, 10 min |  |  |
| Actin | 1095 | 1^st^ amplification | 94 °C, 5 min |  | [9] |
|  |  | F: 5'-ATG(A/G)G(A/T)GAAGAAG(A/T)A(A/G)(C/T)(A/T)CAAGC-3 | 94 °C, 45 sec |  |  |
|  |  | R: 5'- AGAA(G/A)CA(C/T)TTTCTGTG(T/G)ACAAT-3' | 50°C, 45 sec | 35x |  |
|  |  |  | 72°C, 60 sec |  |  |
|  |  |  | 72°C, 10 min |  |  |
|  | 1066 | 2^nd^ amplification | 94 °C, 5 min |  |  |
|  |  | F: 5'- CAAGC(A/T)TT(G/A)GTTGTTGA(T/C)AA-3 | 94 °C, 45 sec |  |  |
|  |  | R: 5'- TTTCTGTG(T/G)ACAAT(A/T)(G/C)(A/T)TGG-3' | 45°C, 45 sec | 35x |  |
|  |  |  | 72°C, 60 sec |  |  |
|  |  |  | 72°C, 10 min |  |  |
|  |  |  |  |  |  |
| COWP | 769 | Amplification | 94°C, 5 min |  | [10] |
|  |  | F: 5´-ACCGCTTCTCAACAACCATCTTGTCCTC-3` | 94°C, 45 sec |  |  |
|  |  | R: 5´-CGC ACC TGT TCC CAC TCA ATG TAA ACC C-3 | 50°C, 45 sec | 30x |  |
|  |  |  | 72°C, 60 sec |  |  |
|  |  |  | 72°C, 10 min |  |  |

**References**

1. Hopkins RM, Meloni BP, Groth DM, Wetherall JD, Reynoldson JA, Thompson RCA. Ribosomal RNA sequencing reveals differences between the genotypes of *Giardia* isolates recovered from humans and dogs living in the same locality. J Parasitol. 1997:83;44-51.

2. Read C, Walters J, Robertson I, Thompson R. Correlation between genotype of *Giardia duodenalis* and diarrhoea. Int J Parasitol. 2002;32:229-31.

3. Sulaiman IM, Fayer R, Bern C, Gilman RH, Trout JM, Schantz PM, et al. Triosephosphate isomerase gene characterization and potential zoonotic transmission of *Giardia duodenalis*. Emerg Infect Dis. 2003;9:1444-52.

4. Read CM, Monis PT, Thompson RCA. Discrimination of all genotypes of *Giardia duodenalis* at the glutamate dehydrogenase locus using PCR-RFLP. Infection, Genet Evol. 2004;4:125-30.

5. Robertson L, Hermansen L, Gjerde B, Strand E, Alvsvåg J, Langeland N. Application of genotyping during an extensive outbreak of waterborne giardiasis in Bergen, Norway, during autumn and winter 2004. Appl Environ Microbiol. 2006;72:2212-7.

6. Cacciò SM, Beck R, Lalle M, Marinculic A, Pozio E. Multilocus genotyping of *Giardia duodenalis* reveals striking differences between assemblages A and B. Int J Parasitol. 2008;38:1523-31.

7. Lalle M, Pozio E, Capelli G, Bruschi F, Crotti D, Cacciò SM. Genetic heterogeneity at the β-giardin locus among human and animal isolates of *Giardia duodenalis* and identification of potentially zoonotic subgenotypes. Int J Parasitol. 2005;35:207-13.

8. Xiao L, Escalante L, Yang C, Sulaiman I, Escalante AA, Montali RJ, et al. Phylogenetic analysis of *Cryptosporidium* parasites based on the small-subunit rRNA gene locus. Appl Environ Microbiol. 1999;65:1578-83.

9. Sulaiman I M, Lal A A, Xiao L. (2002). Molecular phylogeny and evolutionary relationships of *Cryptosporidium* parasites at the actin locus. J Parasitol, 2002; 88:388-394.

10. Yu J R, Lee S U, Park W. Y. Comparative sensitivity of PCR primer sets for detection of *Cryptosporidium parvum*. Korean J Parasitol. 2009;47:293.
